# Paclitaxel loading in PLGA nanospheres affected the *in vitro *drug cell accumulation and antiproliferative activity

**DOI:** 10.1186/1471-2407-8-212

**Published:** 2008-07-25

**Authors:** Luisa Vicari, Teresa Musumeci, Ignazio Giannone, Luana Adamo, Concetta Conticello, Ruggero De Maria, Rosario Pignatello, Giovanni Puglisi, Massimo Gulisano

**Affiliations:** 1 Dipartimento di Oncologia Sperimentale, Istituto Oncologico del Mediterraneo, Viagrande (CT), Italy; 2 Dipartimento di Scienze Farmaceutiche, Università degli Studi di Catania, Catania, Italy; 3 IOM Ricerca S.r.l., Viagrande (CT), Italy; 4 Dipartimento di Ematologia, Oncologia e Medicina Molecolare, Istituto Superiore di Sanità, Roma, Italy; 5 Dipartimento di Scienze Fisiologiche, Università degli Studi di Catania, Catania, Italy

## Abstract

**Background:**

PTX is one of the most widely used drug in oncology due to its high efficacy against solid tumors and several hematological cancers. PTX is administered in a formulation containing 1:1 Cremophor^® ^EL (polyethoxylated castor oil) and ethanol, often responsible for toxic effects. Its encapsulation in colloidal delivery systems would gain an improved targeting to cancer cells, reducing the dose and frequency of administration.

**Methods:**

In this paper PTX was loaded in PLGA NS. The activity of PTX-NS was assessed in vitro against thyroid, breast and bladder cancer cell lines in cultures. Cell growth was evaluated by MTS assay, intracellular NS uptake was performed using coumarin-6 labelled NS and the amount of intracellular PTX was measured by HPLC.

**Results:**

NS loaded with 3% PTX (w/w) had a mean size < 250 nm and a polydispersity index of 0.4 after freeze-drying with 0.5% HP-Cyd as cryoprotector. PTX encapsulation efficiency was 30% and NS showed a prolonged drug release in vitro. An increase of the cytotoxic effect of PTX-NS was observed with respect to free PTX in all cell lines tested.

**Conclusion:**

These findings suggest that the greater biological effect of PTX-NS could be due to higher uptake of the drug inside the cells as shown by intracellular NS uptake and cell accumulation studies.

## Background

PTX is a taxane plant product derived from the bark of the pacific yew tree Taxus brevifolia. Unlike other tubulin binding agents, such as Vinca alkaloids, which induce the disassembly of microtubules, PTX promotes the stabilization of tubulin polymerization [1]. The microtubule formed in the presence of PTX are extremely stable and dysfunctional with a consequent cell cycle arrest at the G2/M phase and inhibition of mitosis [2].

PTX has a significant antineoplastic activity in solid tumor and it is currently approved by both the FDA and the EMEA for the treatment of non-small cell lung cancer, breast cancer, ovarian cancer and AIDS-related Kaposi's sarcoma. In addition PTX is also successfully used, either as single agent and in combination with other antineoplastic agents, in the treatment of thyroid, bladder, head and neck cancers [3-8].

However, the clinical application of PTX is limited by its low therapeutic index and its low solubility in water [9]. Accordingly, standard formulation of PTX (Taxol^®^) requires the use of solvents, such as Cremophor^® ^EL (polyoxyethylated castor oil) and dehydrated alcohol, which contribute to most of the toxicity commonly associated with PTX-based therapy [10]. For these reasons, alternative dosage forms without Cremophor have been proposed, including parenteral emulsions, liposomes, microspheres, cyclodextrin complexes and NP [9,11].

The most interesting approach for PTX drug delivery appears the encapsulation in NP made of biodegradable polymers, such as PLGA or PLA. According to the process in NP preparation, NS or nanocapsules can be obtained. Nanocapsules are vesicular systems in which a drug is confined inside a cavity surrounded by a polymeric membrane whereas NS are matrix systems in which a drug is dispersed through the particles [12]. The encapsulation in NP affects in PTX tissue distribution to cancer cells with a consequent increase of both efficacy and safety [9,13,14]. Furthermore NP, for their small size, can escape from the reticulo-endothelial system and accumulate for passive diffusion inside the tumor; this effect is enhanced by the high vascular permeability and the retention capability typical of the tumor tissue [15]. As a consequence, the higher retention time of intracellular PTX may reduce the number of administrations with a significant improvement of the patient's compliance.

The clinical application of NP-based drug delivery systems, is often limited by the physical (aggregation/particle fusion) and/or chemical instability of the colloidal suspension (hydrolysis of polymer, drug leakage, chemical reactivity of drugs), which become more evident during long term storage [16]. The most efficient technique to improve the stability of these suspension is to remove water, usually by freeze-drying. However, this process generates a variable degree of stress to NP so that protectors are usually added to the formulation [17]. Such protection is usually achieved by adding inert additives, as sugars.

We have recently described the preparation and technological characterization of cryoprotected NS formulations for PTX controlled delivery [18]. One of these formulations, containing HP-Cyd as a cryoprotector, has been further characterized in the present study describing its in vitro activity against the human ARO ATC cell line. Even if ATC is a rare carcinoma, it is one of the most aggressive solid tumors with a mean survival of only six months. ATC are always unresponsive to radioiodine [19] and are therefore treated with a multimodality approach including surgery, radiotherapy or radio-chemotherapy and chemotherapy. Among the newer agent used for chemotherapy, PTX appears the only agent effective on ATC; however PTX is not able to modify in a significant manner the clinical course of such patients [3,20]. The results of our study suggest that the incorporation of PTX in NS enhances its cytotoxic effect on human ARO cells possibly due to higher uptake of the drug inside the cells. These effects have also been confirmed in other two model of human carcinomas, the MDA MB 231 breast carcinoma cells and the RT112 bladder carcinoma cells.

## Methods

### Materials

The following products were commercially available: Resomer^® ^RG 502 H (50:50 PLGA) (Boehringer Ingelheim GmbH, Germany); Tween 80^®^, PTX, PI and Coumarin 6 (Sigma-Aldrich Chimica Srl, Milan, Italy); HP-Cyd (Cyclolab, Budapest, Hungary); RPMI 1640, DMEM, PBS, glutamine, penicillin-streptomycin and FBS (GIBCO, Grand Island, NY, USA); MTS assay (Promega Co., Madison, WI, USA).

ARO cell line was kindly provided by dr. Paolo Vigneri, Department of Biomedical Sciences, University of Catania, Italy; MDA MB231 and RT112 cell line were purchased from the ATCC (American Tissue Culture Collection, Rockville, MD, USA).

All other chemicals and solvents were of analytical reagent grade. De-ionized double-distilled water was used throughout the study.

### Preparation of PTX-loaded NS and coumarin-loaded NS

PLGA NS were prepared by solvent displacement followed by polymer deposition [18]. PTX (3% w/w) and 75 mg PLGA NS were dissolved in 20 ml acetone. The organic phase was poured dropwise (0.5 ml/min), under magnetic stirring, into 40 ml of a water/ethanol solution (1:1, v/v) containing 0.5% (w/v) Tween 80 to obtain a milky colloidal suspension. The organic solvent was then evaporated off under high vacuum at 40°C. The different formulations were purified from untrapped PTX and unadsorbed surfactant by means of centrifugation (15000 × *g*) for 1 h at 5°C, using a Beckman (Fullerton, CA) J2-21 model centrifuge equipped with a Beckman JA-20.01 fixed-angle rotor. The supernatants were discarded and the pellets were resuspended in 50 ml of water, then centrifuged using the same conditions described above. The entire operation was repeated 3 times. After final washing, the NS were resuspended in 5 ml of filtered water (0.22-μm Sartorius membrane filters) in the presence of 5% of HP-Cyd as a cryoprotector [18] and freeze-dried. Freeze-dried NS were resuspended with filtered water and were characterized for size distribution, surface chemistry and technological parameters. For the in vitro studies, freeze-dried NS were resuspended in RPMI 1640 supplemented with 10% heat-inactivated fetal calf serum, 2 mM L-glutamine and 100 U/ml penicillin-streptomycin.

Coumarin 6-loaded NS were prepared similarly for the fluorescent microscopic studies. The polymer solution contained 0.05% (w/v) coumarin-6 as fluorescent marker instead of PTX.

### Physico-chemical, morphological, and technological characterization of PTX-NS

SEM was performed to evaluate the surface morphology of NS using a SEM XL-30 (Philips, Eindhoven, the Netherlands). PTX-NS freeze-dried in the presence and in the absence of the cryoprotector were fixed by means of bi-adhesive tape on a glass disk applied to an aluminum stub (TAAB, Laboratories Equipment, Berks, UK) and evaporated under vacuum overnight. Before the SEM analysis the samples were metallized under argon atmosphere to 10 nm gold palladium thickness (EMITECH-K550 Sputter Coater, Houston, Tex., USA).

NS mean size was determined by PCS using a Zetamaster instrument (Malvern Instruments Ltd, Worcs, England). A solid state laser was used as the light source, with a nominal power of 4.5 mW and a maximum output of 5 mW at 670 nm. The photon correlation spectroscopy measurements were carried out at a scattering angle of 90°. To obtain the mean diameter and polydispersity index of the colloidal suspensions, a third-order cumulant fitting correlation function was performed by a Malvern PCS submicron particle analyzer. For each sample 100 μl were suitably diluted with 2 ml of filtered water to avoid multi-scattering phenomena and placed in a quartz cuvette. The size analysis consisted of 30 measurements per sample, and the results are expressed as mean size ± SD. The following instrument parameters were set: average index of refraction at 1.330, average viscosity at 1.00, and the dielectric constant equal to 79.

Zeta potential distribution was measured with a Zetamaster particle electrophoresis analyzer setup equipped with a 5-mW HeNe laser (633 nm). Limits of ζ-potential ranged from -120 to 120 Volt. Parameters were set as follows: strobe delay -1.00, on time 200.00, off time 1.00. A Smoluchowsky constant F(Ka) of 1.5 was used to achieve ζ-potential values from the electrophoretic mobility data. One hundred μl of each sample were suitably diluted with 20 ml of filtered water.

To assess the drug concentration in the NS, pellets obtained from freeze-dried NS were dissolved in 1 ml of acetonitrile, filtered through 0.45-μm nylon membrane filters (Whatman) and subjected to HPLC analysis Encapsulation efficiency was calculated as the mass ratio of the entrapped drug in NS to the amount used in their preparation.

The in vitro release studies were carried out as described by Mu and Feng [21]. Aliquots of 50 mg of freeze-dried and cryoprotected PTX-loaded NS were placed in a screw-capped tube and suspended in 5 ml of isotonic PBS pH 7.4. The tube was placed in a water bath at 37 ± 0.5°C under magnetic stirring. At fixed time intervals, three tubes for each batch were removed and centrifuged at 14,000 g for 1 h. The supernatants were extracted with 3 × 5 ml-aliquots of dichloromethane. The solvent aliquots were pooled, evaporated under nitrogen and the residue was dissolved in 500 μl of acetonitrile. The resulting solutions were analyzed by HPLC to determine the drug concentration. The pellets were resuspended in 5 ml of fresh PBS and then replaced in the water bath.

The efficiency of the extraction process was evaluated in triplicate using 5 ml of PBS solutions containing different known concentrations of pure PTX and submitted to the same extraction procedure described above, and was found to be 93.9 ± 0.4%.

### Cell lines

ARO human ATC cells, MDA MB 231 human breast carcinoma cells and RT112 human bladder carcinoma cells were grown at 37°C in a 5% CO_2 _atmosphere in RPMI 1640 supplemented with 10% FBS, 2 mM L-glutamine and 100 U/ml penicillin-streptomycin.

### *In vitro* particle cell uptake: fluorescent microscopic studies

ARO cells were seeded in 24-well glass slides. At 80% confluency, medium was replaced with 1 ml of coumarin-6 loaded particle suspension (250 μg/ml). After 2 h of incubation, cells were fixed with 70% ethanol solution and kept at 37°C for 20 min. Subsequently PBS was used to wash wells for three times and 1 μg/ml PI was added to stain cell nucleus for 30–40 min. PI was washed three times using PBS and the glass slides were observed by fluorescence microscopy. Coumarin-6-loaded particles and PI-staining cell nucleus showed green colour and red colour respectively.

### MTS assay and cell viability

Cell viability was evaluated using the MTS colorimetric assay based on the reduction of a tetrazolium compound (MTS) to a colored formazan product that is then measured at 490 nm; absorbance is directly proportional to the number of living cells in the culture. Briefly, cells were seeded in 96-well plates at 5 × 10^3 ^cells/200 μl/well and allowed to adhere to the plate overnight. The next day the cells were treated with free PTX or PTX-NS at a concentration depending on cell type according to its growth curve. Dilutions were made using the culture medium from stock solution of the drug in ethanol or NS suspensions, respectively. After incubation, the culture medium was aspirated and cells were washed with PBS (pH 7.4); 100 μl of fresh culture medium without drug and 20 μl of MTS were added to each well and the cells were incubated for 3 h. The plates were read on a Microplate Reader (Synergy HT, BIO-TEK). Survival was expressed as the percentage of viable cells in treated samples relative to not treated control cells. All the experiments were repeated three times in triplicate.

### Accumulation studies of PTX-NS

Cells were seeded in 6-well plates at 1 × 10^5 ^cells/4 ml/well and allowed to adhere to the plate overnight. After 24 h cells were treated with 2.13 and 4.27 μg/ml of free PTX or PTX NS. After 48 h of incubation, the culture medium was collected and frozen at -80°C (sample A). Adherent cell line were trypsinized; trypsin was inactivated with medium and the suspension was stored at -80°C (sample B).

Sample A containing the culture medium with dead cells was treated as follows: 2.5 ml was incubated with 1.25 ml of ZnSO_4 _(4% w/v in H_2_O/CH_3_OH 70/30) to precipitate proteins. The obtained suspension was vortexed for 5 min, stored at 4°C for 10 min to permit the complete proteins denaturation and then centrifuged at 4000 rpm for 15 min. The supernatant was filtered through 0.2 μm nylon membrane filters (Whatman) and subjected to HPLC analysis to assess the drug concentration (C, amount of intracellular PTX not bound to microtubules and released from NS in the medium). The pellet was treated with 500 μl of CH_3_CN (that was used to break NS and to extract PTX) and centrifuged at 4,000 rpm for 15 min. This procedure was repeated three times to ensure the complete extraction of the drug. The resulting supernatants were dissolved and dried under reduced pressure. The obtained solid samples were dissolved in 500 μl of CH_3_CN, filtered through 0.2 μm nylon membrane filters and analyzed by HPLC to evaluate the amount of PTX not released from NS (B).

Sample B, containing the culture medium with viable cells was treated as follows: cells treated with trypsin were centrifuged at 4,000 rpm for 15 min, the supernatant was eliminated and the pellet was treated with ZnSO_4 _as above described. The solid sample was dissolved with 500 μl of CH_3_CN, filtered and analyzed by HPLC to evaluate the amount of PTX not bound to microtubules (D). The active fraction of PTX (X, bound to tubulin), was calculated as the difference between the initial amount (A) and the total of inactive drug (B, C, D) [X = A - (B + C + D)].

### HPLC analysis

HPLC analysis was performed at room temperature using a 1050 Hewlett Packard apparatus on a 5 μm HP Hypersil ODS cartridge (125 × 4 mm i.d.) equipped with a 5 μm HP Hypersil 100 RP-18 guard cartridge (4 × 4 mm i.d.) and eluted isocratically with acetonitrile/water (60/40, v/v). Flow rate was set at 1 ml/min and UV detection was made at 230 nm. The linear regression coefficient, determined in the range 0.33–33 μg/ml, was 0.9992 (n = 6). The method sensitivity was 3 ng/ml (signal to noise ratio, 3:1).

### Statistical analysis

All data were analyzed by using Student t-test. A p value less than 0.05 was accepted as statistically significant. All data analyses were done using Minitab statistical software (Origin Lab ver. 7.0, ORIGINLAB Co., Northampton, MA, USA).

## Results

**Figure 1:**
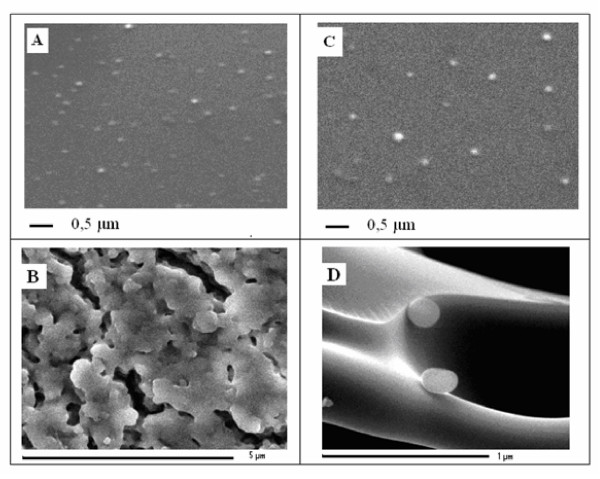
**Scanning electron microscopy of 3% PTX-NS**. Scanning electron micrographs of PTX-NS without cryoprotector (1A), freeze-dried without cryoprotector (1B), with the addition of HP-Cyd as a cryoprotector (1C) and freeze-dried with HP-Cyd (1D). Magnifications: 1 μm (A and D), 0.5 μm (B), 5μm (C).

The morphological characteristics of PTX NS freeze-dried in the presence or absence of the cryoprotector HP-Cyd have been examined by SEM (Figure 1). Before freeze-drying, the presence of HP-Cyd did not influence the mean size and the homogeneity of the NS since they maintained a smooth and spherical surface (Figures 1A and 1C). By contrast, the absence of cryoprotector caused evident signs of aggregation, that hindered the following resuspension (Figure 1B); the presence of HP-Cyd prevented the aggregation of NS (Figure 1D). These results confirm therefore the importance of a cryoprotector to maintain the physico-chemical parameters of a colloidal suspension for long term storage.

**Figure 2:**
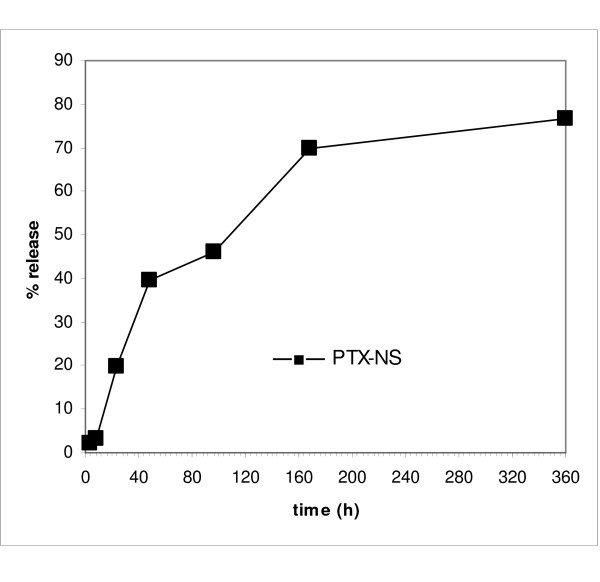
***In vitro* release of PTX-NS**. PTX 3% loaded NS in pH 7.4 phosphate buffer solution, at 37°C for 360 h. Data are expressed as the mean ± SD of five separate experiments.

Mean particle size, polydispersity index, Zeta potential, and encapsulation efficiency of PTX-NS with HP-Cyd before and after freeze-drying are shown in Table 1. All the parameters were slightly modified after freeze-drying but without any significant difference (data not shown); with respect to empty NS, PTX loading did not altered them in a significant manner. The in vitro PTX release from PLGA NS displayed a rapid leakage during the first 2 days and a constant release which was completed within 15 days (Figure 2).

**Table 1:** Mean particle size, size distribution (polidispersivity index, PDI), Zeta potential and encapsulation efficiency (EE) of empty PLGA NS and PTX-NS before and after freeze-drying.

	**Freeze-drying**	**Mean size (nm)**	**PDI**	**Zeta potential (mV)**	**% E.E.**
PLGA NS	before	292.20 ± 72.50	0.385 ± 0.144	-5. 20 ± 0.10	-
	after	386.76 ± 45.50	0.340 ± 0.150	-4. 60 ± 0.20	

PTX-NS	before	192.00 ± 18.40	0.296 ± 0.071	-6.20 ± 0.20	35.59 ± 2.53
	after	286.76 ± 45.50	0.340 ± 0.089	-5.10 ± 0.10	

Data are expressed as the mean ± SD of five separate experiments.

### 2. Biological activity of PTX-NS in ARO thyroid cancer cells

**Figure 3:**
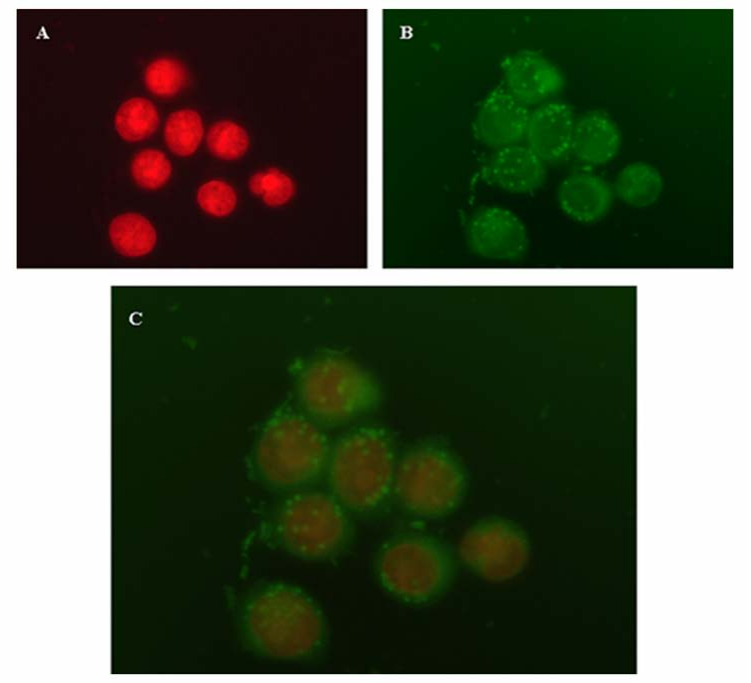
**Intracellular distribution of NS in ARO cells**. ARO cells were incubated for 2 h with PLGA NS loaded with the fluorescent coumarin-6 probe and analyzed by fluorescent microscopy. Nuclei were stained with PI and are visible in red (1A). The uptake of coumarin-6-loaded NS is visible in green (1B). Figure 1C displays an overlaying images obtained combining the FITC and the PI filters. A representation of two experiments is shown. Magnification: 63 ×

We first studied the ability of ARO cells to uptake the PLGA NS. Cells were incubated for 2 h with PLGA NS loaded with the fluorescent (FITC) probe Coumarin-6 and then examined by fluorescent microscopy. Nuclei were stained with PI. No fluorescence was detected from cells not exposed to Coumarin-6 proving the absence of cell or NS auto-fluorescence (data not shown). The fluorescence of Coumarin-6 loaded PLGA NS (green) was closely located around the nuclei (red) indicating a significant accumulation of PLGA NS by ARO cells (Figure 3).

**Figure 4:**
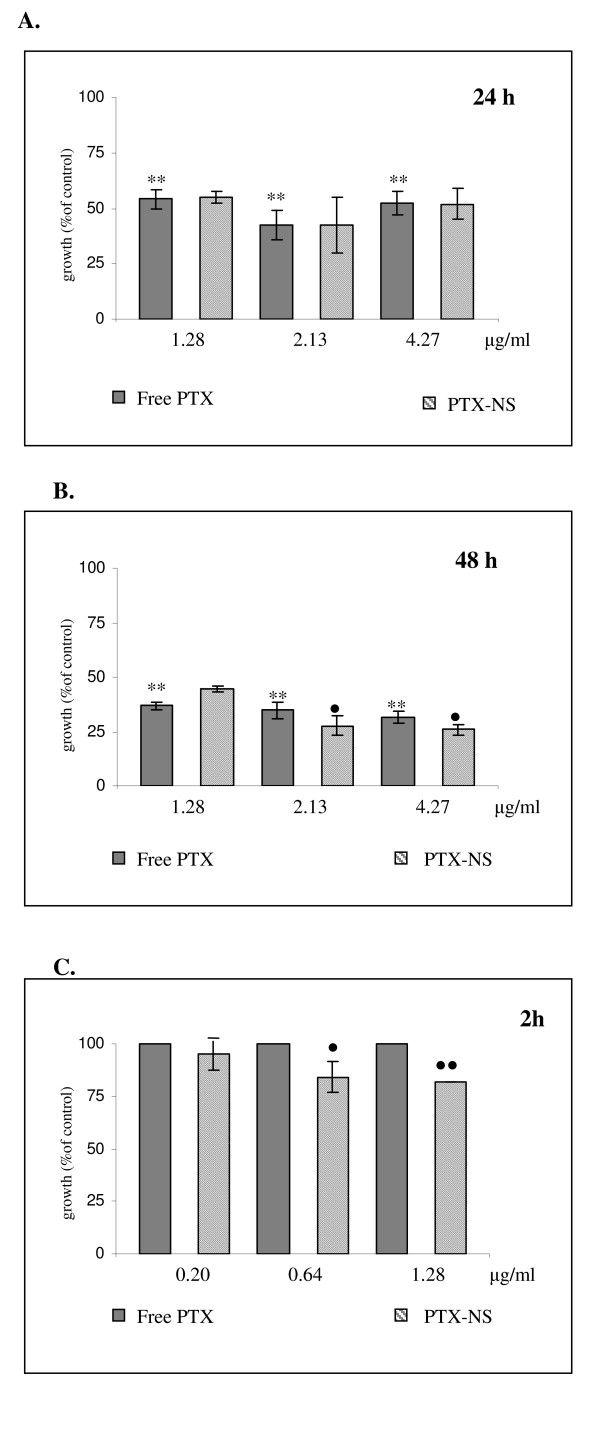
**Cytotoxicity of free PTX and PTX-NS in ARO cells**. ARO cells were treated with increasing concentrations (0.2 – 4.27 μg/ml) of either free or NS-loaded PTX for different times (4A = 24 h, 4B = 48 h, 4C = 2 h). Cell viability was assessed by the MTS assay. Data are expressed as % of viable cells (mean ± SD of three separate experiments, each performed in triplicate) exposed to PTX in respect to untreated controls. ^• ^free PTX vs. PTX-NS, *treated vs. not treated ^•^*p < 0.05 ^••^**p < 0.01

The cytotoxicity and subsequent therapeutic potential of PTX-NS was thereafter evaluated by incubating ARO cells with increasing concentrations of free or NS-loaded PTX (1.28 to 4.27 μg/ml) for 24 and 48 h. Cell growth was then assessed by using the MTS assay. Empty PLGA NS were not cytotoxic to cells at all concentrations and time interval tested (data not shown). PTX reduced cell growth in a significant manner (p < 0.01, Student's t test) by approximately 40% and 70% after 24 and 48 h of exposure, respectively; a clear dose-response relationship was not observed in both experimental conditions (Figure 4A and 4B). The two formulations (free PTX and PTX-NS) had a comparable cytotoxic effect after 24 h of exposure whereas, after 48 h, PTX-NS was more potent than free PTX starting at a concentration of 2.13 μg/ml (p < 0.05). Interestingly, we observed that PTX-NS was also capable to induce a significant (p < 0.05) decrease of cell growth (approximately 20%) under the experimental conditions (2-h incubation and PTX concentrations below 1.0 μg/ml) in which free PTX was not active (Figure 4C).

**Figure 5:**
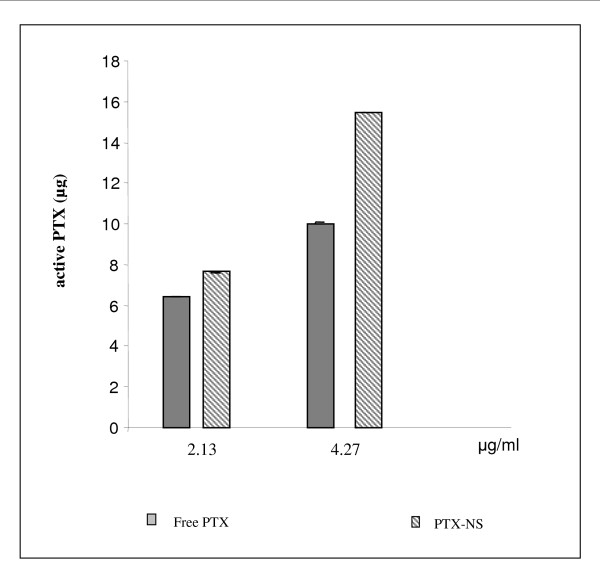
**Accumulation of PTX in ARO cells**. ARO cells were incubated for 48 h with 2.13 or 4.27 μg/ml of either free PTX or PTX-NS. The amount of active PTX present in the cells was analyzed by HPLC. Data are expressed as mean ± SD of three replicates.

Finally, in order to investigate whether the observed higher cytotoxic effect of PTX-NS in respect to free PTX was due to higher intracellular uptake of PTX, the amount of intracellular PTX bound to tubulin (active PTX) was measured by HPLC (accumulation studies). For this purpose, ARO cells were exposed to either free PTX or PTX-NS at concentrations able to inhibit cell growth in a significant manner (2.13 μg/ml and 4.27 μg/ml); at the end of incubation (48 h) cells were processed and active PTX quantized. The percentage of active PTX was approximately 90% of the loaded dose for PTX-NS and only 60 to 70% for free PTX (at 4.27 and 2.13 μg/ml, respectively). Accordingly, the absolute amount of PTX bound to tubulin was higher after incubation with PTX-NS with a 2-fold difference in respect to the free PTX at the concentration of 4.27 μg/ml (Figure 5).

### 3. Biological activity of PTX-NS in other cell lines

To prove that our findings were not confined to thyroid cells, the cytotoxic activity PTX-NS was assessed against other cell lines derived from tumor types in which PTX is effective, such as breast and bladder carcinomas.

**Figure 6:**
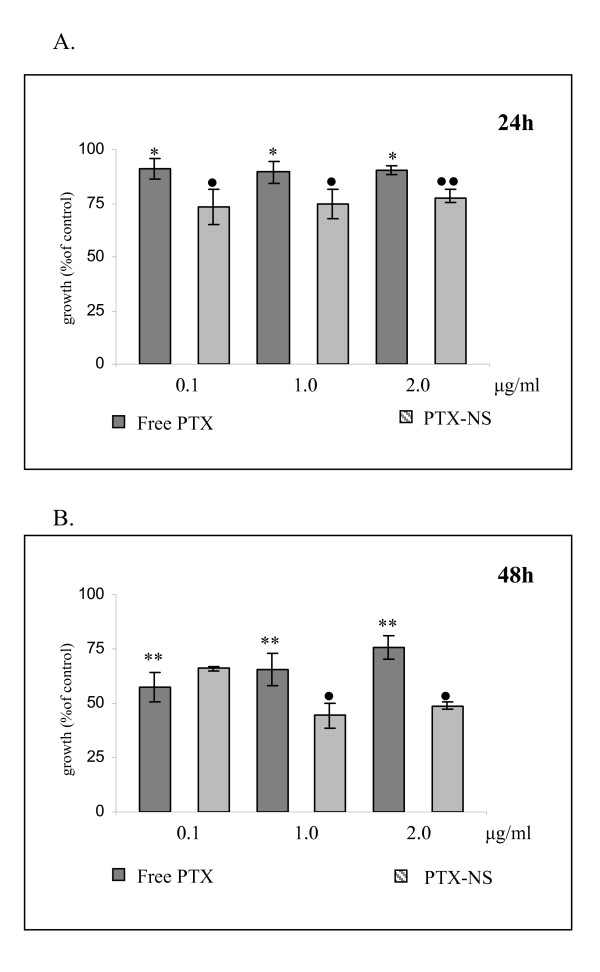
**Cytotoxicity of free PTX and PTX-NS in MDA MB231 cells**. MDA MB231 cells were treated with increasing concentrations (0.1 – 2.0 μg/ml) of either free PTX or PTX-NS for 24 (6A) or 48 (6B) h and cell viability assessed by the MTS assay. Data are expressed as % of viable cells (mean ± SD of three separate experiments, each performed in triplicate) exposed to PTX in respect to untreated controls. ^• ^free PTX vs. PTX-NS, *treated vs. not treated *p < 0.05 ^••^**p < 0.01

**Figure 7:**
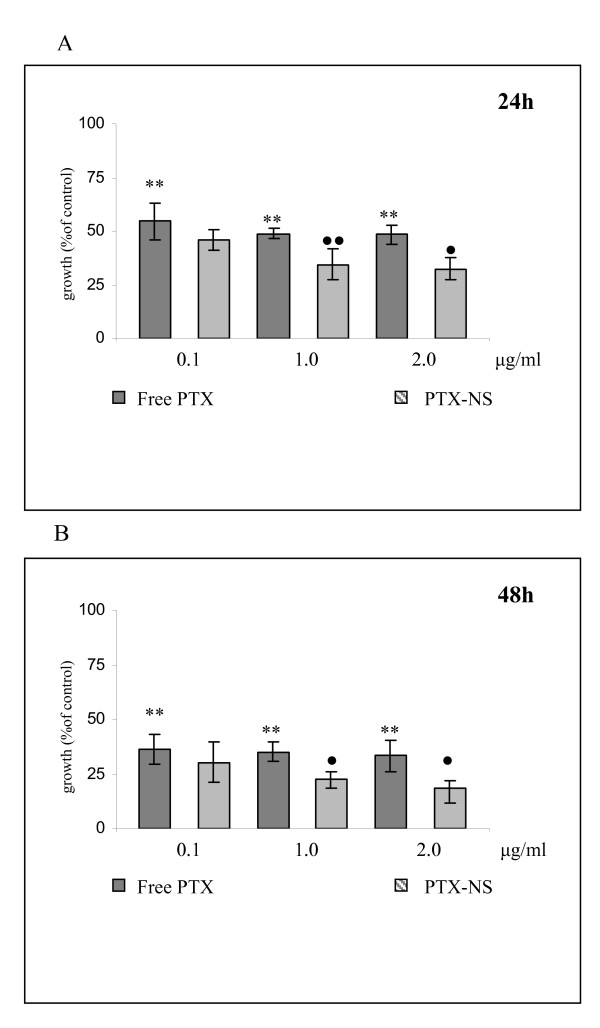
**Cytotoxicity of free PTX and PTX-NS in RT 112 cells**. RT 112 cells were treated with increasing concentrations (0.1 – 2.0μg/ml) of either free PTX or PTX-NS for 24 (7A) or 48 (7B) h and cell viability was assessed by the MTS assay. Data are expressed as % of viable cells (mean ± SD of three separate experiments, each performed in triplicate) exposed to PTX in respect to untreated controls. ^• ^free PTX vs. PTX-NS, *treated vs. not treated ^•^*p < 0.05 ^••^**p < 0.01

Data obtained in MDA MB 231 breast cancer cells are shown in Figure 6 whereas data from RT112 bladder cancer cells are shown in Figure 7 (panels A = 24 h, panels B = 48 h). Empty PLGA NS was not toxic to cells even at the higher concentrations (data not shown) and cell growth decreased significantly even at the lower concentration of free PTX tested (0.1 μg/ml) in a time dependent manner but without a clear dose-response relationship. PTX-NS had a more powerful activity at all doses but reached a statistical significance at the concentration of 1 μg/ml. Among cells, RT112 bladder cancer cell line was the most responsive to PTX with a 80% maximal inhibition of cell growth.

## Discussion

PTX is one of the most interesting drugs currently available for cancer therapy. Its primary place in therapy is in the treatment of breast, ovarian and lung (non-small-cell) cancers and AIDS-related Kaposi's sarcoma, but it is also used in other malignancies, including ATC, bladder, head and neck cancers [3-8]. However the clinical utility of PTX is limited by its poor water solubility and therefore systemic administration of this drug relies upon concomitant use of Cremophor EL^® ^to produce an adequately soluble formulation. Unfortunately, Cremophor EL^® ^use is also associated with patient toxicity as it is not well tolerated and leads to hypersensitivity reactions in some individuals. To overcome these difficulties, clinicians have attempted to prolong infusion schedules or use corticosteroids and anti-histaminic drugs as a part of a pre-medication regimen. However, to improve the efficacy of PTX in anti-cancer therapy, a potential solution must involve reformulation of the drug into better-tolerated and selectively delivered vehicles. Accordingly, a number of strategies to develop alternative formulations of PTX are in progress, including the use of albumin NP, pro-drugs (Xyotax and Taxoprexin), co-solvents (Genexol PM), emulsions, liposomes and microspheres [22].

Among these new drug delivery systems, polymeric NP are considered the interesting carriers for PTX as well as other anticancer agents. In fact NP are easily prepared with biodegradable polymers (as PLGA) and are highly stable in biological fluids and during storage [9,18]. In addition these systems are known to distribute to tumor sites to a great extent due to a passive targeting phenomenon which takes advantage of the enhanced permeability and retention effect [23]. The hyper-permeability of tumor tissue vasculature allows the extravasations of NP within tumor interstitium and the concomitant lack of tumor lymphatic drainage in the tumor bed, resulting in NP accumulation. As a consequence NP are able to increase the concentration of the carried chemotherapeutic agent within the tumor tissue [23]. For instance, FDA has recently approved Ambraxane as the first protein-stabilized NP formulation of PTX for its significantly improved cytotoxic activity in respect to the standard PTX formulation.

The most common drawback of clinical application of NP is their thermodynamic instability of their suspension that require water removal, usually by the freeze-drying technique. This step, however, can negatively affect the colloidal system and therefore an adjuvant, commonly called cryoprotector, is usually added to the solution with the aim to preserve the morphology of colloidal particles and their following reconstitution in water [17]. We have previously described the encapsulation of PTX in NS by a solvent displacement followed by polymer deposition method to evaluate the effect of different polymers and cryoprotectors on particle diameter, size distribution and drug loading capacity [18]. We found that this method produces stable PTX-NS which can be stored for a long period without loosing their physical and chemical characteristics upon reconstitution [18]. A colloidal NS suspension containing PLGA and HP-Cyd, has been further characterized in the present study. PLGA has been chosen because it is one of the most suitable FDA approved biodegradable polymer; HP-Cyd, on the other hand, is a very effective cryoprotector [18] which is able to maintain physical characteristics of the colloidal suspension as demonstrated by the SEM studies conducted herein.

The used PLGA produced small NS (~300 nm) with a low polydispersity index, indicating a homogeneous size distribution. After freeze-drying, NS size slightly increased (to about 400 nm), most probably because of the absorption of the cryoprotector. PTX-loaded NS showed a smaller size than unloaded particles; this effect can be due to a simultaneous precipitation of polymer and PTX in the aqueous phase, with a consequent reduction of the amount of polymer per particle. Accordingly, also the polydispersity index and the zeta potential values were lower for PTX-NS in respect to the unloaded NS. It is important to underline the close relationship between NS size and biological effect of PTX since smaller particles are able to better pass through the leaky endothelial cells [23]. The amount of PTX released from the NS in which it is uniformly embedded was approximately 75% of the loaded dose. Such release generally occurs by diffusion or erosion of the matrix and depends on different parameters (for example particle size and/or molecular weight of the polymer). The release behaviour of the formulation used in this study showed a biphasic pattern, with an initial relevant burst effect (possibly due to the diffusion of the co-precipitated drug from NS surface), followed by a slower and more constant release. Similar patterns have been described for the release of PTX from other polymeric systems [9,11,24].

An ATC cell line (ARO cells) was used in this study as the main model to determine the anti-tumor activity of PTX, either free or incorporated in NS. Some data have also been replicated on two other cell lines derived of tumor types in which PTX is effective, namely breast cancer (MDA MB 231 cells) and bladder carcinoma (RT112 cells) indicating that our findings are not confined to thyroid cells. Even if ATC is a rare carcinoma, it is one of the most aggressive and lethal solid tumors known to affect humans with a median survival of 4 to 12 months from the time of diagnosis. Because > 50% of ATC patients have metastastic disease at presentation, the importance of chemotherapy in the management of ATC cannot be understated [20]. However, outcomes with chemotherapy have been disappointing with a few patients with partial responses and almost none with a complete response after either mono-therapy or combination therapy [20]. It has been shown that PTX has some effect on ATC patients but is not capable to alter the course of the disease [3]. These data suggest the need for additional therapeutic innovations, such as the encapsulation of drugs in colloidal carriers able to an increase the drug content inside the neoplastic cells [9,12,23,25].

When the biologic activity of free and PTX-NS have been compared, we observed analogies and differences between the two systems.

Regarding the biologic effects of PTX-NS two main differences were observed in respect to free PTX, one in terms of efficacy and the other of kinetics.

In all the three cellular model tested in this study, the cytotoxicity of PTX-NS was higher than that of free PTX. RT 112 cancer cells were the most sensitive to both forms, whereas MDA MB 231 breast cancer cells were the most resistant ones.

A different mechanism of transport of PTX-NS in respect to the free drug can be claimed to explain such differences. In fact, drug encapsulation in PLGA NS caused a rapid internalization of the system inside the cells by endocytosis, resulting in a higher cellular uptake. Once inside the cells, NS escaped the endo-lysosomal pathway and entered the cytoplasm where they are retained for a longer time [26].

As a further difference, the kinetic profile of the cytotoxic activity of PTX-NS was more rapid. PTX-NS significantly inhibited ARO cells growth after only 2 h of incubation, whereas the effect of free PTX started after 24 h. These results can be related to the fast internalization of PTX-NS, that ensure a quicker and higher drug concentration available inside the cells. On the other hand, also our coumarin-6 uptake studies confirmed such a trend.

The goal of our work was to determine the amount of intracellular (active) PTX. Actually its application was limited because of the difficulty in determining the intracellular PTX fraction bound to tubulin. To the best of our knowledge this is the first quantitative demonstration by HPLC that a NS delivery system may significantly increase the amount of available PTX inside cells, suggesting that the greater biological effect of PTX-NS could be due to its efficient internalization and sustained retention inside the cells.

## Conclusion

In summary, a methodology to produce small and homogeneous NS exhibiting high incorporation efficiency of PTX was described. The incorporation of PTX in PLGA NS enhanced its cytotoxic activity in three different in vitro tumor models responsive to PTX, due to a higher cell uptake of the drug, that we were able to demonstrate.

Even if these data need to be confirmed in other systems (such as human primary culture or tumor xenografts), this formulation appears as an interesting delivery system for PTX in the treatment of cancer.

## Abbreviations

3-(4,5-dimethylthiazol-2-yl)-5-(3-carboxymethoxyphenyl)-2-(4-sulfophenyl)-2H-tetrazolium, inner salt: MTS; anaplastic thyroid cancer: ATC; CellTiter 96 AQ_ueous_One solution Cell Proliferation Assay: MTS assay; fetal bovine serum: FBS; hydroxypropyl-β-cyclodextrin: HP-Cyd; nanoparticles: NP; nanospheres: NS; Paclitaxel: PTX; phosphate buffered saline: PBS; photon correlation spectroscopy: PCS; poly D,L-lactide: PLA; Poly(D,L-lactide-*co*-glycolide): PLGA; polyoxyethylene sorbitan monoleate: Tween 80; propidium iodide: PI; scanning electron microscopy: SEM.

## Competing interests

The authors declare that they have no competing interests.

## Authors' contributions

LV designed and performed most of the biological experiments, analyzed and interpreted most of the data and drafted the manuscript; TM performed chemical and technological experiments and revised the manuscript; IG performed HPLC analysis and data interpretation and help to revise the manuscript; LA performed fluorescent microscopic studies; CC contributed to data analysis and revised the manuscript; RDM contributed to a final revision of the version to be submitted. RP contributed to the design of experiments and data interpretation and help to revise the manuscript; GP provided a critique of the experiments on the technical aspects, and contributed to manuscript revision; MG conceived the project and assisted in data interpretation as well as undertaking the final revision of the manuscript. The order of the authors was arranged accordingly.

## Pre-publication history

The pre-publication history for this paper can be accessed here:


